# Strong antimicrobial activity and unique physicochemical characteristics in honey from Australian stingless bees *Tetragonula carbonaria*, *Tetragonula hockingsi*, and *Austroplebeia australis*

**DOI:** 10.1128/aem.02523-24

**Published:** 2025-05-21

**Authors:** Kenya E. Fernandes, Aviva Levina, Nural N. Cokcetin, Dean Haley, Jasmin Li, Peter Brooks, Rosalyn Gloag, Dee A. Carter

**Affiliations:** 1 School of Life and Environmental Sciences, University of Sydney 4334, Sydney, New South Wales, Australia; 2 Sydney Institute for Infectious Diseases, University of Sydney 4334, Sydney, New South Wales, Australia; 3 School of Chemistry, University of Sydney 217058, Sydney, New South Wales, Australia; 4 Australian Native Bee Association, Brisbane, Queensland, Australia; 5 School of Science, Technology and Engineering, University of the Sunshine Coast 276017, Sippy Downs, Queensland, Australia; Anses, Maisons-Alfort Laboratory for Food Safety, Maisons-Alfort, France

**Keywords:** honey, *Tetragonula carbonaria*, *Tetragonula hockingsi*, *Austroplebeia australis*, antimicrobial activity, natural products, hydrogen peroxide, phenolics, stingless bees, native bees

## Abstract

Natural products have evolved antimicrobial properties that can be exploited in our search for new ways of treating infectious diseases. This study evaluates the antimicrobial properties and chemical profiles of honey produced by the Australian stingless bee species *Tetragonula carbonaria*, *Tetragonula hockingsi*, and *Austroplebeia australis* against selected human pathogens. Using broth microdilution methods, we found that all tested honey samples had antimicrobial activity. The fungal dermatophyte species *Trichophyton interdigitale* displayed the highest susceptibility (average MICs: 4%–9% [wt/wt]), followed by *Staphylococcus aureus* (9%–11%), *Escherichia coli* (10%–13%), and *Cryptococcus neoformans* (24%–34%). *T. carbonaria* honey had the highest overall activity, while *A. australis* honey was the weakest. After heat treatment at 80°C for 30 min to remove hydrogen peroxide (H_2_O_2_)-based activity, *T. carbonaria* and *T. hockingsi* honey retained significant non-peroxide activity against *E. coli* (14% and 17%, respectively) and *S. aureus* (17% and 18%, respectively), although their efficacy against the fungal pathogens diminished. Chemical analysis revealed distinct differences in H_2_O_2_ production, color intensity, phenolic and antioxidant content, density, and pH among the honey types. The dynamic generation of H_2_O_2_ in stingless bee honey was remarkably prolonged, with some samples producing H_2_O_2_ for more than 6 days. Proteomic analysis identified diverse proteins that may contribute to antimicrobial efficacy. Phenolic extracts had antimicrobial activity, with flavonoids identified as potential contributors. *T. carbonaria* honey re-tested after 18 years of storage retained substantial non-peroxide-based activity. Overall, this study highlights the unique properties of stingless bee honeys and their potential as natural antimicrobial agents.

**IMPORTANCE:**

Antimicrobial resistance poses a critical global health challenge. Various natural products have evolved as a defense against microbial attack and can be exploited as novel therapeutic agents. While honey from the European honey bee (*Apis mellifera*) is well studied, the medicinal potential of Australian stingless bee honey remains underexplored. This study demonstrates that honeys produced by the stingless bee species *Tetragonula carbonaria*, *Tetragonula hockingsi*, and *Austroplebeia australis* possess unique antimicrobial properties that persist after heat treatment and following long-term storage and are distinct from the antimicrobial properties of honey bee honey. Diverse bioactive compounds, including phenolics and proteins, were seen, underscoring the complexity of these honeys as antimicrobial agents. These honeys have potential as sustainable, natural solutions for combating drug-resistant infections and could extend the scope of honey-based therapeutics.

## INTRODUCTION

Limitations of the current arsenal of antimicrobials and the global rise of antimicrobial resistance pose a critical public health threat that is driving the search for natural alternatives (1). Honey from the European honey bee (*Apis mellifera*) has long been valued for its medicinal properties and has re-emerged in modern medicine as a promising candidate due to its broad-spectrum antimicrobial, anti-inflammatory, and antioxidant effects (2). However, while the medicinal properties of *A. mellifera* honey are well established, a significant knowledge gap exists regarding the potential of honey from other bee species, such as Australian stingless bees (Meliponini).

In Australia, native stingless bee species like *Tetragonula carbonaria*, *Tetragonula hockingsi*, and *Austroplebeia australis* produce a distinct honey, known locally as “sugarbag” (3). These bees, found in tropical and subtropical regions, have held an important place in Indigenous Australian culture for thousands of years (4). Their honey has not only served as a nutritious food source but has also been used in traditional medicine to treat itchy skin and sores and as a holistic remedy that balances both physical and spiritual health (5). Despite this rich cultural history, scientific research on the antimicrobial properties of Australian stingless bee honey remains limited. Initial studies on *Tetragonula* honey have indicated promising antimicrobial activity (6–9); however, the potential of honey from other Australian stingless bees, such as *Austroplebeia*, is yet to be explored.

The antimicrobial activity of honey can generally be classified into two types: peroxide activity and non-peroxide activity. Peroxide activity arises from the enzymatic production of hydrogen peroxide (H_2_O_2_) through the enzyme glucose oxidase (10), while non-peroxide activity refers to antimicrobial properties that do not rely on H_2_O_2_ production, often linked to other bioactive compounds such as phenolics and flavonoids. A well-known example of non-peroxide activity is in Manuka honey, attributed to methylglyoxal (MGO) derived from the nectar of *Leptospermum* plants. Previous studies have identified both peroxide activity and non-peroxide activity in Australian *Tetragonula* honey (6) and in honey from other neotropical stingless bee species (11, 12). Stingless bee honey differs from *A. mellifera* honey in several key aspects, including higher moisture content (~30% compared to the ~20% maximum for *A. mellifera* honey [13]), lower pH, and distinctive flavor profiles (14). However, the mechanisms underlying antimicrobial activity in stingless bee honey, and how they compare to *A. mellifera* honey, remain poorly understood.

In this study, we comprehensively evaluate the antimicrobial activity of honey produced by *T. carbonaria*, *T. hockingsi*, and *A. australis* against selected bacterial and fungal human pathogens and analyze the composition of this honey, including H_2_O_2_ production and protein and phenolic content, to provide new insights into the mechanisms underlying its bioactivity. We highlight the ways in which Australian stingless bee honey is unique from that produced by honey bees, advancing our understanding of it as a valuable natural antimicrobial agent that could contribute to the fight against antimicrobial resistance.

## MATERIALS AND METHODS

### Honey samples and preparation

A total of 48 honey samples were collected from colonies of *T. carbonaria* (*n* = 20), *T. hockingsi* (*n* = 20), and *A. australis* (*n* = 8) between 2021 and 2024 (one sample per colony). An additional set of older *T. carbonaria* (*n* = 6) honey samples previously studied by Irish et al. in 2008 and stored in plastic containers in the dark at 4°C was used in some analyses (6). Details of all honey samples are presented in Table S1. Honey samples collected fresh from colonies were centrifuged at 3,000 g for 5 min to separate and remove pollen and other debris. Following arrival in the lab, all samples were stored in plastic containers in the dark at 4°C. Controls used in various tests included artificial honey (ART; 1.5 g sucrose, 7.5 g maltose, 40.5 g fructose, 33.5 g glucose, and 17 mL sterile water), Barnes TA 10 + *A. mellifera* jarrah honey (JAR) with H_2_O_2_-based activity, and Comvita UMF 18 + *A. mellifera* (MAN) with non-peroxide based activity. Unless otherwise specified, all honey samples were mixed with a spatula, diluted to the target concentration in sterile water, and vortexed thoroughly before use.

### Microorganisms and culture conditions

One gram-positive bacterium (*Staphylococcus aureus* ATCC29213), one gram-negative bacterium (*Escherichia coli* ATCC25922), one yeast (*Cryptococcus neoformans* H99), and one mold (*Trichophyton interdigitale* CTI1) were used for antimicrobial testing. Bacterial and yeast strains were maintained as glycerol stocks at –80°C. *T. interdigitale* was maintained as agar cuts in water. Bacterial strains were grown on nutrient agar (NA; Oxoid) and incubated at 30°C for 24 h before use. Fungal strains were grown on potato dextrose agar (Oxoid) and incubated at 30°C for 48 h (*C. neoformans*) or for up to 7 days until good sporulation was obtained (*T. interdigitale*).

### Antimicrobial susceptibility testing

The phenol equivalence assay was performed according to Irish et al. (15). Antimicrobial susceptibility testing by broth microdilution was performed in accordance with Clinical and Laboratory Standards Institute (CLSI) guidelines for aerobic bacteria (16), yeasts (17), and filamentous fungi (18) with minor modifications. Briefly, inocula were prepared from colonies growing on agar plates to a final concentration of 2 × 10^5^ to 8 × 10^5^ CFU/mL for bacteria, 0.5 × 10^3^ to 2.5 × 10^3^ colony forming units CFU/mL for *C. neoformans*, and 1 × 10^3^ to 3 × 10^3^ CFU/mL for *T. interdigitale*. Bacterial strains were adjusted to an absorbance of between 0.08 and 0.1 at 540 nm, while fungal strains were counted using a hemocytometer.

Assays used tryptone soya broth (Oxoid) for bacteria, yeast nitrogen base (Sigma-Aldrich) supplemented with 0.165 M morpholinepropanesulfonic acid (MOPS) and 0.5% D-glucose for *C. deuterogattii*, or RPMI-1640 (Sigma-Aldrich) supplemented with 0.165 M MOPS and 2% D-glucose for *T. interdigitale*. For heat-treated activity testing, honey was diluted to 64% (wt/wt), heated to 80°C for 30 min, and allowed to return to room temperature before testing. Honeys were assayed at 1%–32% (wt/wt) diluted in sterile water. Phenolic extracts were assayed at 1%–256% relative to their concentration in honey. Tetracycline was included as a drug control for bacterial strains and amphotericin B as a drug control for fungal strains.

Plates were incubated without agitation at 35°C for 20 h (bacteria), 48 h (*C. neoformans*), or 96 h (*T. interdigitale*). The MIC was determined visually and defined as the lowest honey concentration at which growth was completely inhibited (no visible turbidity). Three independent repeats were performed for each honey sample.

### Hydrogen peroxide assays

H_2_O_2_ production was measured in honey samples using peroxide test strips (Precision Laboratories Cat. No. IS124-50S). For untreated samples, a small amount of honey was applied to test strips directly using a wooden toothpick. For dilution treatment, honey was diluted to 25% (wt/wt) and allowed to accumulate H_2_O_2_ before measurement at various time points. For 35°C heat treatment, honey was heated for 72 h before measurement. For 80°C heat treatment, honey was diluted to 50% (wt/wt), heated for 30 min, then allowed to return to room temperature before measurement. Test strips were allowed to develop for 10 seconds for 25% and 50% (wt/wt) samples, or for 1 min for 100% (wt/wt) samples, to allow honey to fully penetrate the strip material. Results were read and compared to the color chart provided by the manufacturer to determine H_2_O_2_ concentration.

For time-course measurements, samples were diluted to 25% (wt/wt), and H_2_O_2_ concentration was measured using test strips at 1 h, 12 h, 24 h, and every subsequent 24 h for 6 days. A scanner was used to capture an image of the test strips. This was converted to grayscale (image > type > 16 bit) in ImageJ (version 1.52a National Institutes of Health, USA), and the grayscale intensity values from 0 to 255 were measured (analyze > set measurements > mean gray value). Known concentrations of H_2_O_2_ were used to generate a standard curve to convert the greyscale intensity data back to parts per million (ppm).

### Physical and chemical property assays

The Fast Blue BB (FBBB) assay for measuring phenolic content and the ferric-reducing antioxidant power (FRAP) assay for measuring antioxidant content were performed as previously described in Fernandes et al. (19). For color intensity, honey samples were diluted to 50% (wt/wt) in MilliQ water, and absorbance at 450 and 720 nm was measured using a UV/Vis spectrophotometer (Specord S600) in disposable plastic cuvettes with 10 mm optical pathlength. Color intensity was then calculated using the equation (*A*_450_ − *A*_720_) × 1,000 and expressed as milli absorbance units (mAU). For density, honey samples were diluted to 50% (wt/wt) in MilliQ water, and the weight of 1 mL of this solution was measured to 0.001 mg using an analytical micro balance (Shimadzu AUW220D). Density was then calculated using the equation [weight (g) − 0.5 g] × 1,000 and expressed as gram per milliliter. For pH, 10% (wt/wt) honey samples were measured using a pH meter (Mettler Toledo) at room temperature. For the determination of methylglyoxal, dihydroxyacetone (DHA), and hydroxymethylfurfural content, honey samples were derivatized with O-(2,3,4,5,6-pentafluorobenzyl)hydroxylamine HCl and analyzed against anisole by high-performance liquid chromatography (HPLC) with 263 nm detection (20).

### Proteomic analysis

Samples were prepared following a modified version of the protein extraction protocol described in Bocian et al. (21). Initially, 100 µL of honey was dissolved in an equivalent volume of Milli-Q water, followed by precipitation using a chloroform/methanol cleanup. This method was chosen over the acetone-based extraction described by Bocian et al. (21) due to improved solubility of the samples. The resulting precipitate was resuspended in 100 µL of a urea/thiourea solution (6M/2M), and the protein concentration was quantified using a Qubit assay. For each sample, 20 µg was reduced with dithiothreitol and alkylated with iodoacetamide, prior to digestion with porcine trypsin at a 1:50 enzyme-to-protein ratio and overnight incubation at 37°C.

Peptides were concentrated and desalted using Oasis HLB 10 mg cartridges following the manufacturer’s instructions, then resuspended in 20 µL of 3% (vol/vol) acetonitrile/0.1% (vol/vol) formic acid. Peptide separation was carried out via nano-LC using a Neo Vanquish HPLC and autosampler system (Thermo Fisher Scientific) coupled to an in-house built fritless nano-column (75 µm × 30 cm) containing ReproSil Pur 120 C18 stationary phase (1.9 µm, Dr. Maisch GmbH). LC mobile phase buffers comprised buffer A (0.1% [vol/vol] formic acid) and buffer B (80% [vol/vol] acetonitrile/0.1% [vol/vol] formic acid). Peptides were eluted using a linear gradient of 5%–30% buffer B over 77 min, followed by a 98% buffer B wash for 15 min at a flow rate of 300 nL/min.

For LC analysis, the LC was coupled to an Orbitrap Eclipse mass spectrometer (Thermo Fisher Scientific) with a FAIMS unit operating with a compensation voltage of −50. The electrospray voltage was set to 2,300 V, and the heated capillary was set to 300°C. Full mass spectrometry scans (MS1) were acquired in positive ion mode over a mass range of 350–1,650 m/z at a resolution of 60,000, with a maximum injection time of 50 ms and maximum AGC set to 200%. For MS/MS, the AGC target was set at 5.0E4, the isolation window was set to 1.6 m/z, the collision energy was set to 30, and dynamic exclusion was set for 15 s.

Data were analyzed using Proteome Discoverer 2.5 (Thermo Fisher Scientific) and Mascot version 2.8 (Matrix Science, London). Search parameters included the following variable modifications: acetylation (protein N-terminus), oxidized methionine, deamination (asparagine and glutamine), and carbamidomethylation (cysteine). Trypsin was specified as the enzyme, allowing for up to two missed cleavages, with a 10 ppm precursor mass tolerance and 0.05 Da product ion mass tolerance. The data were searched against the publicly available Apidae database (NCBI Taxonomy ID: 7458) and a custom in-house contaminants database.

### Isolation and characterization of phenolic extracts

Phenolic fractions of honey from *T. carbonaria* (sample TC19), *T. hockingsi* (sample TH14), and *A. australis* (sample AA07) were isolated by solid phase extraction with hydrophobic Amberlite XAD-2 resin (Merck Cat. No. 10357) using a modified literature protocol (22). The yield of phenolic extracts was calculated relative to the original honey samples as the weight percentage (wt/wt). For instance, approximately 18.4 g of TC06 honey yielded 53 mg of methanolic extract, making 2.9 mg of extract per gram equivalent to an extract concentration of 100% (relative to honey). ^1^H nuclear magnetic resonance (NMR) spectra of phenolic extracts (~5 mg/mL in d^6^-DMSO) were collected on a Bruker Avance 500 MHz spectrometer at 300 K, using a standard ^1^H NMR setup (a sum of 64 scans), and processed by MestReNova Lite-14.0.0–23239 (MestReLab Research) and OriginPro 2022 (OriginLab) software. The spectra were calibrated and normalized using the DMSO residual peak at 2.50 ppm. Full details of the solid phase extraction protocol, NMR, and liquid chromatography-mass spectrometry (LC-MS) characterization are outlined in Material S3; Fig. S1.

### Honey microbial content

After thorough mixing, sterile cotton swabs were immersed in undiluted honey samples, and the honey was then spread evenly across the entire surface of NA plates. Plates were incubated at 35°C for 5 days and photographed using a digital camera. Two independent replicates were performed on different days.

### Statistical analysis

Whether data were normally distributed was determined using the Shapiro-Wilk normality test. For statistical analysis, MIC readings above the tested maximum of 32% (wt/wt) honey were assigned a value of 64%. Differences between groups were analyzed by one-way analysis of variance (ANOVA), followed by Tukey’s post-hoc test for multiple comparisons. For correlation analyses, all MIC values were reversed so that variables related to increased antimicrobial activity would appear as positively rather than negatively correlated. Associations between antimicrobial activity and the physical and chemical properties of honey samples used Spearman’s rank correlation, which determines the strength and direction of monotonic relationships. *P* values < 0.05 were considered significant. Data were analyzed using Prism 10.0.0 (GraphPad Software).

## RESULTS

### Stingless bee honeys have high levels of total activity and retain some potency after heat treatment

**Fig 1:**
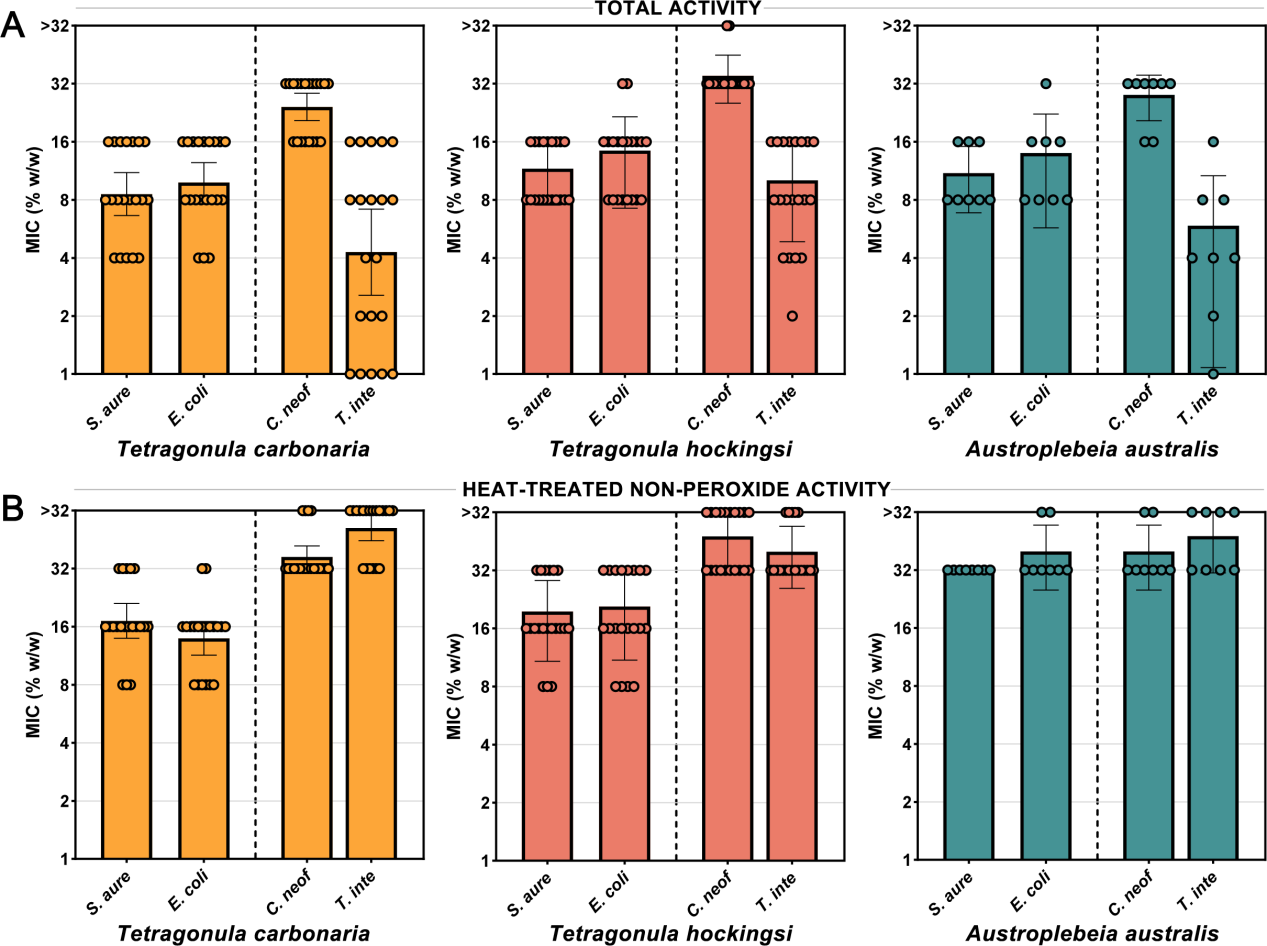
Total and heat-treated non-peroxide activity of stingless bee honey against bacteria and fungi. (**A**) Total and (**B**) heat-treated non-peroxide activity MICs of honey samples from *T. carbonaria* (*n* = 20), *T. hockingsi* (*n* = 20), and *A. australis* (*n* = 8) against bacterial pathogens *S. aureus* and *E. coli* (left columns) and fungal pathogens *C. neoformans* and *T. interdigitale* (right columns). The % (wt/wt) refers to the concentration of honey diluted in sterile water. For heat-treated non-peroxide activity, honey was diluted to 64% (wt/wt), heated to 80°C for 30 min, and allowed to return to room temperature before testing. Columns represent the geomean while circles show the values of individual honey samples. Error bars show the 95% confidence interval.

The antimicrobial activity of a collection of 20 *T*. *carbonaria*, 20 *T. hockingsi*, and 8 *A*. *australis* honey samples was assessed using broth microdilution methodology against gram-positive (*S. aureus*), gram-negative (*E. coli*), yeast (*C. neoformans*), and mold (*T. interdigitale*) human pathogens (Table 1; Fig. 1). All honey tested had detectable antimicrobial activity. Considering the total activity (Fig. 1A) of honey from the three bee species, *T. interdigitale* and *S. aureus* were the most susceptible microbes with average MICs ranging from 4%–9% and 9%–11% (wt/wt), respectively. *E. coli* (10%–13%) was significantly less susceptible (*P* ≤ 0.004) than both *T. interdigitale* and *S. aureus*, while *C. neoformans* (24%–34%) was significantly less susceptible than all other organisms tested (*P* < 0.001). Overall, for total activity, *T. carbonaria* honey was significantly more active than *T. hockingsi* honey against *C. neoformans* (*P* = 0.026). No other significant differences were seen.

**TABLE 1:** All antimicrobial, physical, and chemical data for Australian stingless bee honey samples

Sample ID	MIC (% wt/wt)^*a*^								Physical and chemical properties					
	*S. aureus*		*E. coli*		*C. neoformans*		*T. interdigitale*		H_2_O_2_ at 1 h (ppm)	Color intensity (mAU)	Phenolics content by FBBB (mg GAE/kg)^*a*^	Antioxidant content by FRAP (µmol Fe^2+^/kg)	Density (g/mL)	pH
	TA	HT	TA	HT	TA	HT	TA	HT						
*T. carbonaria*														
TC01	8	16	16	32	32	>32	16	32	35	191	221	8,909	1.26	3.85
TC02	4	16	8	16	16	32	2	>32	60	381	398	9,620	1.26	3.73
TC03	8	16	16	16	32	32	4	>32	55	294	235	7,043	1.25	3.77
TC04	8	32	8	16	16	32	8	>32	65	144	382	5,895	1.26	3.66
TC05	16	32	16	16	32	>32	8	>32	40	163	346	7,571	1.25	3.68
TC06	4	16	4	8	16	32	1	>32	65	215	209	7,240	1.23	3.53
TC07	4	16	4	8	16	32	1	>32	55	144	303	8,860	1.23	3.50
TC08	16	32	16	32	32	32	8	32	45	171	166	6,142	1.22	3.60
TC09	16	32	16	16	32	32	4	32	60	166	177	7,480	1.22	3.61
TC10	4	16	4	16	16	32	1	>32	60	335	232	6,965	1.24	3.75
TC11	8	8	8	8	32	32	16	>32	40	103	162	10,226	1.26	3.59
TC12	4	16	8	16	16	32	1	>32	60	136	151	10,057	1.27	3.60
TC13	16	16	16	16	32	32	16	32	30	651	340	9,304	1.26	3.60
TC14	16	16	16	16	32	>32	8	>32	50	261	365	9,142	1.28	3.62
TC15	16	16	16	16	32	>32	16	32	40	662	308	9,261	1.30	3.60
TC16	8	32	8	8	32	32	2	>32	50	252	233	11,184	1.26	3.54
TC17	8	16	8	16	16	32	2	>32	75	248	314	7,930	1.35	3.72
TC18	16	16	16	16	32	32	16	32	5	196	104	5,804	1.31	3.59
TC19	8	8	8	8	16	32	1	>32	55	295	261	10,796	1.27	3.64
TC20	8	8	8	8	32	32	8	>32	45	272	227	9,902	1.26	3.65
**Mean^*b*^**	**9**	**17**	**10**	**14**	**24**	**37**	**4**	**52**	**50**	**264**	**257**	**8,467**	**1.26**	**3.64**
*T. hockingsi*														
TH01	16	16	16	16	32	>32	16	32	40	298	238	9,613	1.34	3.82
TH02	8	8	8	8	32	>32	4	>32	50	455	521	9,529	1.31	3.71
TH03	8	8	8	8	32	>32	8	>32	50	464	432	9,057	1.36	3.72
TH04	8	16	8	32	32	>32	8	32	65	490	511	9,649	1.30	3.91
TH05	16	16	16	32	32	32	16	32	45	393	312	11,388	1.36	3.87
TH06	8	16	8	32	32	>32	2	32	70	497	439	9,895	1.36	3.96
TH07	16	16	16	16	32	>32	4	32	55	257	716	11,416	1.25	3.74
TH08	16	16	16	16	>32	>32	8	32	45	266	365	11,501	1.35	3.72
TH09	8	16	8	8	32	>32	4	>32	55	464	615	10,458	1.39	3.66
TH10	16	32	16	32	32	32	16	32	45	272	273	8,923	1.40	3.85
TH11	8	32	16	32	32	32	8	32	60	470	648	9,395	1.28	3.95
TH12	16	32	32	32	32	32	16	32	60	282	304	9,684	1.28	3.93
TH13	16	32	16	32	32	>32	16	32	40	300	357	8,346	1.33	3.88
TH14	8	32	8	16	32	32	8	32	55	485	478	8,261	1.29	3.99
TH15	16	16	16	16	32	32	8	32	60	214	111	2,965	1.29	3.83
TH16	8	16	8	16	32	32	16	>32	30	294	144	4,599	1.30	3.72
TH17	8	8	16	8	32	32	16	>32	45	362	237	6,635	1.29	3.67
TH18	16	16	32	16	>32	>32	16	32	30	217	103	5,923	1.28	3.96
TH19	8	16	16	16	32	32	8	32	60	258	258	8,289	1.27	3.68
TH20	8	32	8	32	32	32	4	32	50	266	261	7,106	1.27	3.72
**Mean^*b*^**	**11**	**18**	**13**	**18**	**34**	**45**	**9**	**38**	**51**	**350**	**366**	**8,632**	**1.31**	**3.81**
*A. australis*														
AA01	16	32	16	32	32	32	8	32	35	507	224	6,458	1.33	3.72
AA02	8	32	8	32	16	32	1	32	45	220	88	4,282	1.31	4.76
AA03	16	32	16	>32	32	>32	8	>32	20	392	182	7,423	1.31	3.85
AA04	8	32	8	32	16	32	4	32	50	107	10	4,698	1.28	3.82
AA05	8	32	8	32	32	32	4	32	50	511	193	4,867	1.30	4.19
AA06	8	32	8	32	32	>32	2	32	40	115	118	3,367	1.29	4.36
AA07	16	32	32	>32	32	32	16	>32	40	429	163	5,071	1.28	4.26
AA08	8	32	16	32	32	32	4	>32	45	380	251	2,613	1.282	4.06
**Mean^*b*^**	**10**	**32**	**12**	**38**	**27**	**38**	**4**	**42**	**41**	**333**	**154**	**4,848**	**1.30**	**4.13**

^*a*^ TA = total activity and HT = heat treated (diluted to 64% [wt/wt] and heated to 80°C for 30 min).

^*b*^ Geomean is shown for MIC data (bold values). For MIC values >32, a value of 64 was assigned for the purposes of calculating geomean.

To remove H_2_O_2_ activity, honey was diluted to 64% (wt/wt), then heated to 80°C for 30 min and allowed to return to room temperature. Non-peroxide activity is typically measured using catalase to neutralize H_2_O_2_; however, initial results showed that catalase itself affected the growth of certain microbes, prompting us to use heat treatment as an alternative approach to remove H_2_O_2_ activity. Since non-peroxide activity may involve bioactive compounds that could be sensitive to heat, the heat-treated samples may have undergone alterations beyond just the removal of H_2_O_2_. Consequently, we refer to this as “heat-treated non-peroxide activity” rather than specifically non-peroxide activity.

Results for heat-treated non-peroxide activity (Fig. 1B) were more variable compared to total activity. Heat-treated *T. carbonaria* honey had significantly stronger (*P* < 0.001) average activity against the bacteria *E. coli* (14%) and *S. aureus* (17%) compared to the fungi *C. neoformans* (37%) and *T. interdigitale* (52%). Heat-treated *T. hockingsi* honey followed a similar pattern with significantly stronger (*P* < 0.001) average activity against bacteria *S. aureus* (18%) and *E. coli* (18%) compared to fungi *T. interdigitale* (38%) and *C. neoformans* (45%). Heat-treated *A. australis* honey had the weakest activity against both bacteria and fungi, with low-level average activity against *S. aureus* (32%), *E. coli* (38%), *C. neoformans* (38%), and *T. interdigitale* (42%) and no significant differences in susceptibility across microbes. To confirm that the non-peroxide activity present in the heat-treated honey was not due to MGO, which is the main contributor to the non-peroxide activity of manuka honey, three *T. carbonaria*, three *T. hockingsi*, and one *A. australis* sample were analyzed by HPLC. All samples contained <5 ppm of MGO and 0 ppm of DHA, the precursor to MGO (Table S1). Overall, heat-treated *T. carbonaria* and *T. hockingsi* honey was significantly more active against bacteria (*P* ≤ 0.001) than *A. australis* honey, and *T. carbonaria* honey was significantly more active against *T. interdigitale* (*P* = 0.011) than *T. hockingsi* honey.

### Stingless bee honeys are distinct from each other in their physical and chemical properties

**Fig 2:**
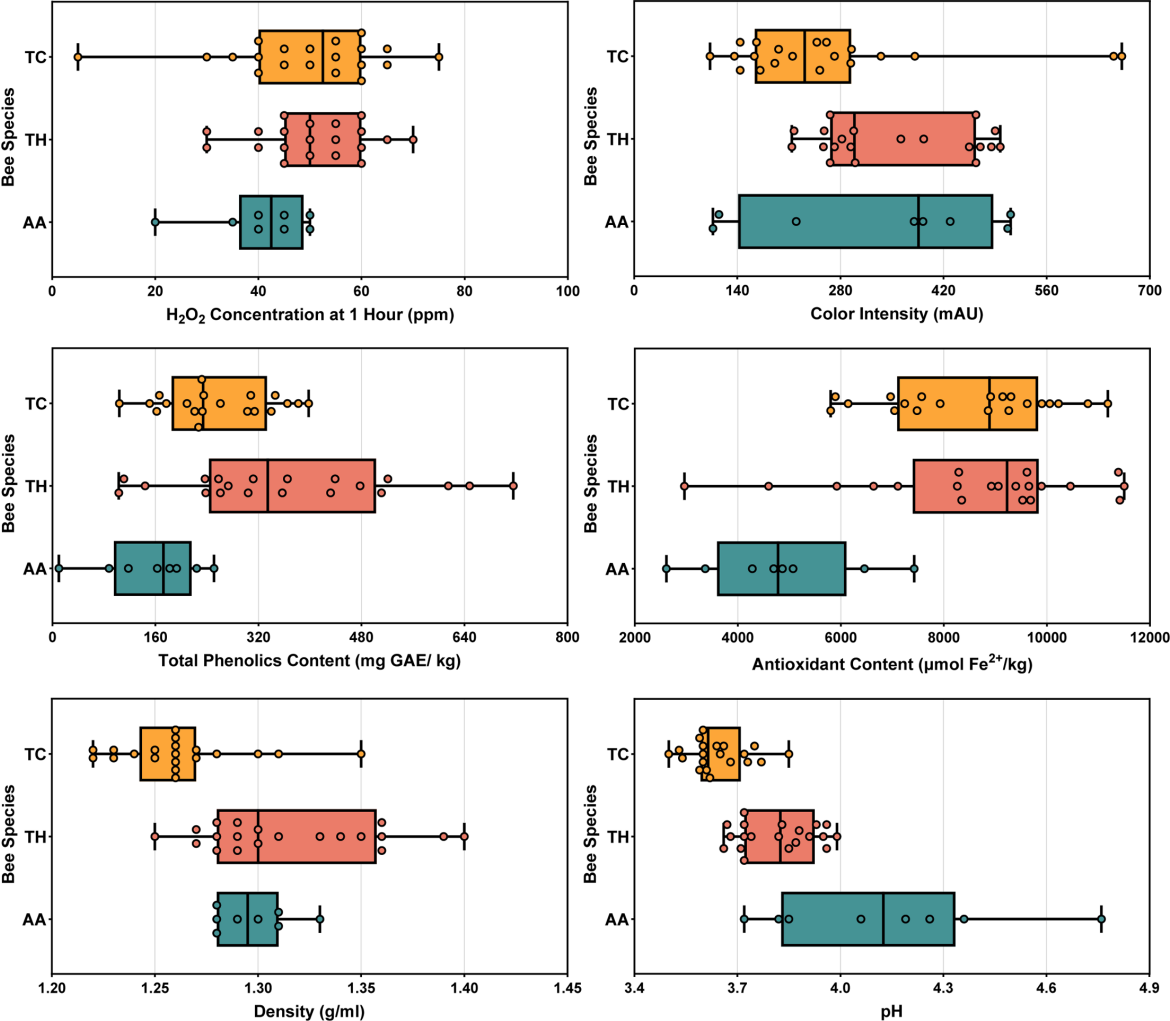
Physical and chemical properties of stingless bee honey. H_2_O_2_ concentration at 1 h, color intensity, total phenolics content, antioxidant content, density, and pH of honey samples from *T. carbonaria* (TC; *n* = 20), *T. hockingsi* (TH; *n* = 20), and *A. australis* (AA; *n* = 8). Box plot lines show the 25th percentile, the median, and the 75th percentile, with whiskers extending to the minimum and maximum values.

Physical and chemical properties of stingless bee honeys that may contribute to their antimicrobial activity were measured and compared (Table 1; Fig. 2). For H_2_O_2_ concentration, *T. carbonaria* honey had the highest median at 53 ppm, followed closely by *T. hockingsi* honey at 50 ppm, and *A. australis* honey substantially lower at 43 ppm. For color intensity, *A. australis* honey had the highest median at 386 mAU, with *T. hockingsi* and *T. carbonaria* honeys at 299 and 232 mAU, respectively, though the highest overall values were obtained by two *T. carbonaria* honeys (651 and 662 mAU). Due to the high level of variation among samples within each honey type, there were no statistically significant differences among means or variance for either H_2_O_2_ concentration or color intensity.

For phenolic content, *T. hockingsi* honey had the highest median at 335 mg GAE/kg, followed by *T. carbonaria* honey at 234 mg GAE/kg (*P* = 0.029), and *A. australis* honey at 173 mg GAE/kg. *T. hockingsi* honey had significantly greater average phenolic content (*P* ≤ 0.029) as well as significantly more variance (*P* = 0.003). A similar pattern was evident for antioxidant content, with *T. hockingsi* honey having the highest median at 9,226 µmol Fe^2+^/kg, followed by *T. carbonaria* honey at 8,885 µmol Fe^2+^/kg, and *A. australis* honey at 4,782 µmol Fe^2+^/kg. In this case, *A. australis* honey had significantly lower median antioxidant content (*P* ≤ 0.001) but no significant differences in variance.

For density, *T. hockingsi* honey had the highest median at 1.30 g/mL and the widest range of values (1.25–1.4 g/mL), with the median of *A. australis* honey close behind at 1.29 g/mL and *T. carbonaria* honey lower at 1.26 g/mL. *T. hockingsi* honey had significantly greater average density (*P* ≤ 0.001) compared to *T. carbonaria* honey as well as significantly more variance (*P* = 0.046). For pH, *A. australis* honey had the highest median at 4.13, with *T. hockingsi* and *T. carbonaria* honeys substantially lower at 3.83 and 3.62, respectively. *A. australis* honey had significantly higher average pH (*P* ≤ 0.001) and significantly more variance (*P* ≤ 0.001) than both *T. hockingsi* and *T. carbonaria* honeys, with *T. carbonaria* honey, in turn, having significantly higher average pH than *T. hockingsi* honey (*P* = 0.005).

### Stingless bee honey exhibits distinct H_2_O_2_ generation dynamics compared to honey bee honey

**Fig 3:**
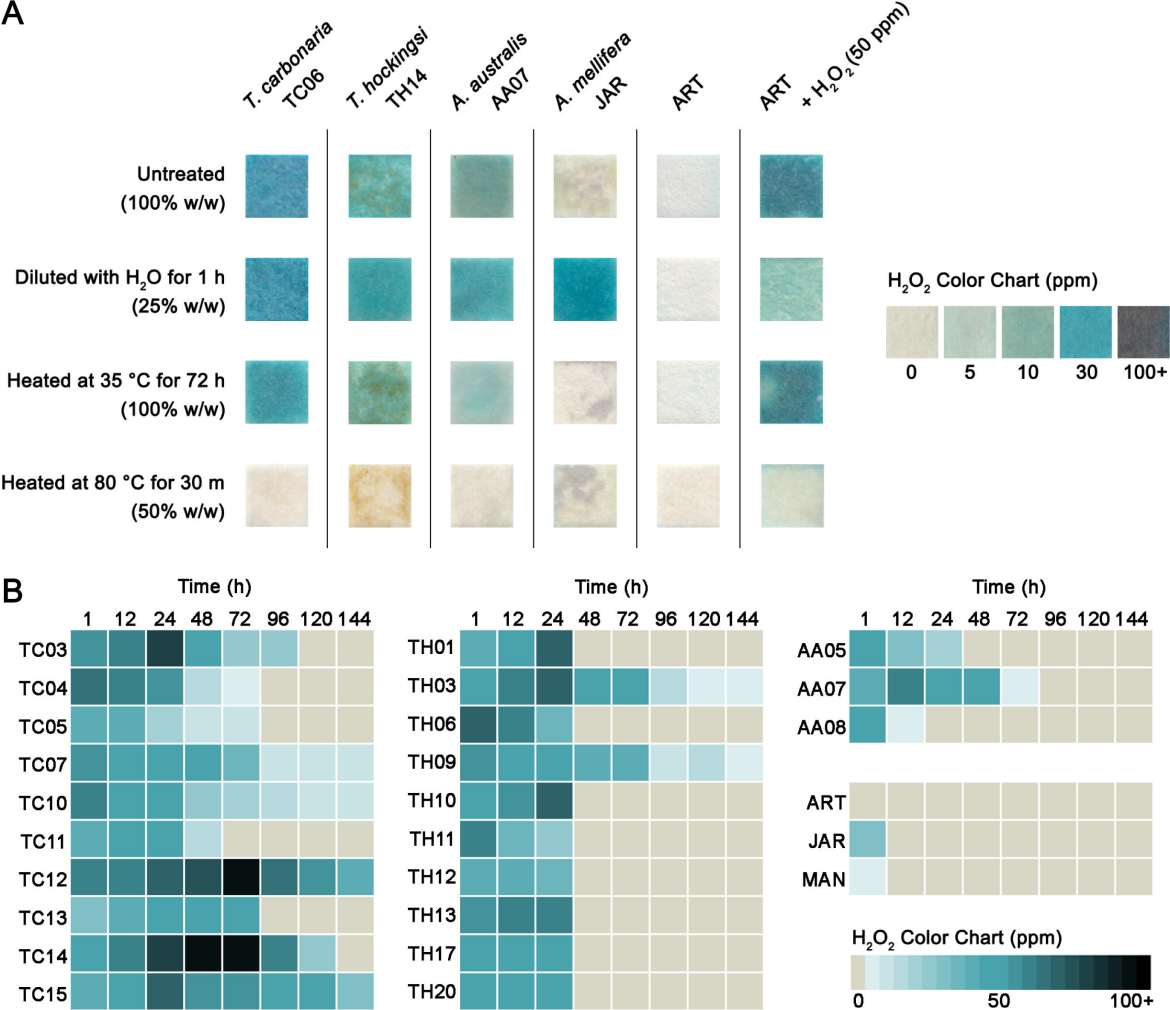
H_2_O_2_ dynamics in stingless bee honey. H_2_O_2_ production in stingless bee honey measured using peroxide test strips. (**A**) For undiluted honey, samples were applied to test strips directly using a wooden toothpick. For diluted honey, samples were diluted to 25% (wt/wt) using sterile water and allowed to accumulate H_2_O_2_ for 1 h before measurement. For 35°C heat treatment, honey was heated for 72 h before measurement. For 80°C heat treatment, honey was diluted to 50% (wt/wt) using sterile water, heated for 30 min, then allowed to return to RT before measurement. *A. mellifera* jarrah honey (JAR), which has a known high level of H_2_O_2_ production and peroxide-type activity, and artificial honey (ART) alone or spiked with 50 ppm H_2_O_2_ were included for comparison. (**B**) Time course measurements of H_2_O_2_ production and accumulation in stingless bee honey over a period of 144 h. ART, high peroxide-type activity JAR and high non-peroxide-type activity MAN were included for comparison.

The enzyme glucose oxidase, which produces H_2_O_2_ in honey bee honey, is inactive in undiluted honey, in part due to the viscosity of the solution. Due to the relatively higher water content in stingless bee honey compared to honey bee honey, we hypothesized that the H_2_O_2_ production dynamics might be different. To test this, one honey sample from each stingless bee species (TC06, TH14, and AA07) was applied undiluted to peroxide test strips using a wooden toothpick (Fig. 3A). The three undiluted stingless bee honey samples displayed 10–30 ppm of H_2_O_2_, while *A. mellifera* jarrah honey (which is known to produce high levels of H_2_O_2_ when diluted [23]) displayed none, giving a similar result to the artificial honey control. When samples were diluted to 25% (wt/wt) and allowed to accumulate H_2_O_2_ for 1 h, all three stingless bee honey samples and the *A. mellifera* jarrah honey displayed around 30 ppm of H_2_O_2_. To test the stability of H_2_O_2_ at elevated temperatures, undiluted honey samples were heated at 35°C for 72 h. The three stingless bee honeys retained H_2_O_2_ concentrations similar to those seen prior to heating, while *A. mellifera* jarrah honey again displayed none. Finally, samples were diluted to 50% (wt/wt) and heated to 80°C for 30 min, which should denature any enzyme present and decompose H_2_O_2_. The absence of H_2_O_2_ was confirmed in all samples.

When assessed spectrophotometrically over time, H_2_O_2_ production in diluted honey bee honey normally exhibits an “inverted U-shaped” curve (23, 24). The maximal peak of this curve varies depending on the sample and usually occurs around 2–3 h post-dilution, dropping to zero by ~24 h. To determine the strength and duration of H_2_O_2_ production in stingless bee honey, H_2_O_2_ concentration was measured using the peroxide test strips at 1 h, 12 h, 24 h, and every subsequent 24 h for 6 days in 10 *T. carbonaria*, 10 *T. hockingsi*, and 3 *A. australis* honeys (Fig. 3B). Control *A. mellifera* jarrah honey gave 30 ppm of H_2_O_2_ at 1 h and returned to 0 by 12 h. In contrast, the stingless bee honey produced H_2_O_2_ over a more extended period. *T. carbonaria* honey exhibited by far the strongest and longest H_2_O_2_ production, with extremely high production over days in some samples and the total time of production ranging from 48 to >144 h. H_2_O_2_ production for most *T. hockingsi* honey samples returned to undetectable levels by 48 h; however, in two samples, this continued for >144 h. *A. australis* honey samples had a shorter duration of H_2_O_2_ production than the other stingless bee samples, ranging from 12 to 72 h, but these were still longer than the *A. mellifera* jarrah honey.

### Correlations between antimicrobial activity and physical and chemical properties

**Fig 4:**
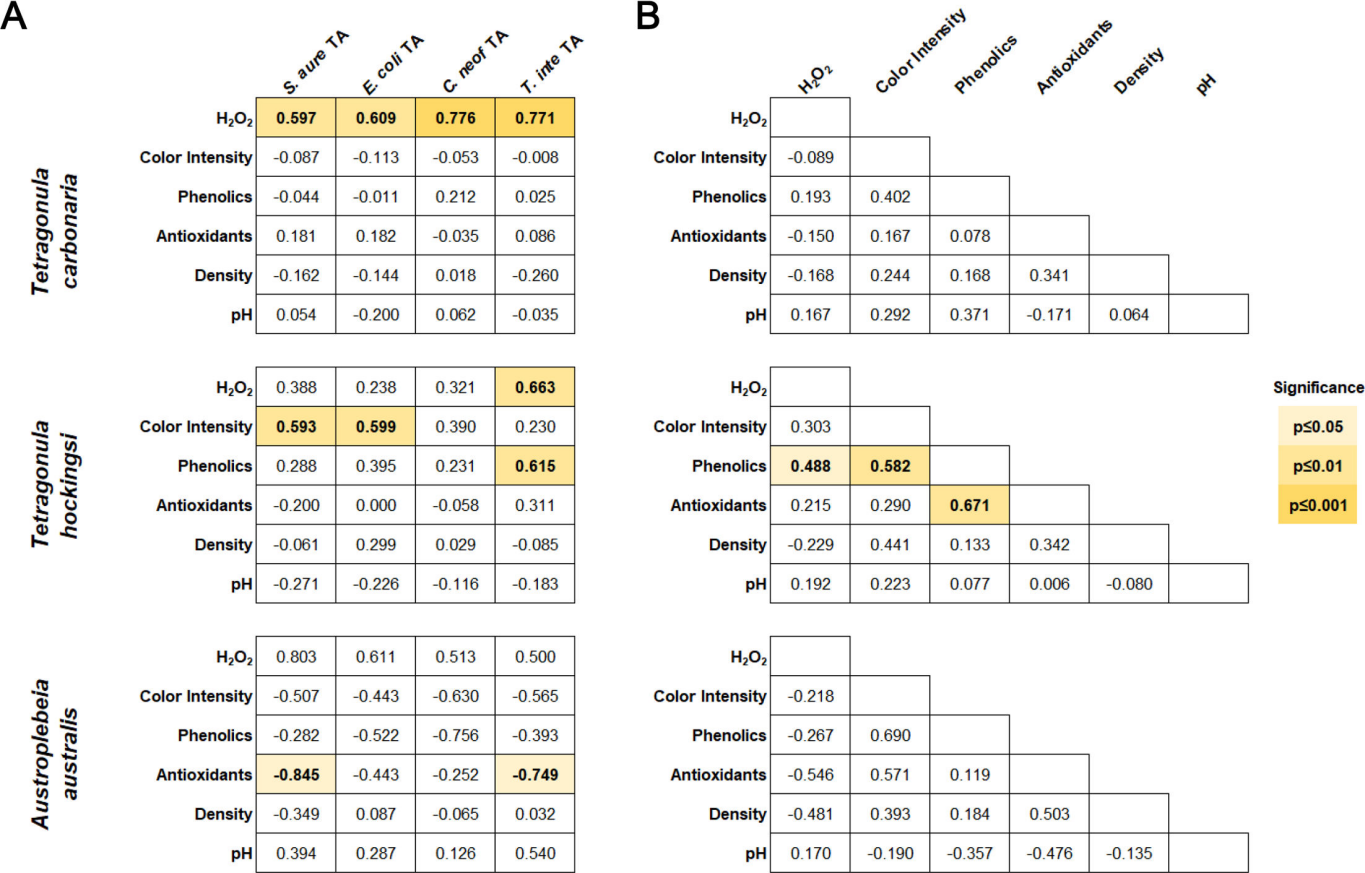
Correlations between antimicrobial activity and the physical and chemical properties of stingless bee honey. Spearman correlation matrices show how closely and in what direction (**A**) antimicrobial activity (horizontal) aligns with physical and chemical properties (vertical), and (**B**) physical and chemical properties align with each other. MIC values have been reversed so that positive correlations indicate properties that align with increased antimicrobial activity. Sperman’s rho values are shown in the boxes with 1 indicating the strongest positive correlation and −1 indicating the strongest negative correlation. Statistically significant correlations are shaded in yellow with the corresponding significance level indicated in the legend to the right.

A Spearman correlation matrix was generated to assess the strength and direction of associations between antimicrobial activity data and physical and chemical property data (Fig. 4A). For this, MIC values were reversed so that variables related to increased antimicrobial activity would appear as positively correlated. For *T. carbonaria* honey, H_2_O_2_ was significantly correlated with activity against all four organisms (*P* ≤ 0.01). For *T. hockingsi* honey, color intensity was significantly correlated with activity against *S. aureus* (*P* ≤ 0.01) and *E. coli* (*P* ≤ 0.01), suggesting that compounds that darken the honey play a significant role in its antibacterial effect. H_2_O_2_ (*P* ≤ 0.01) and phenolic content (*P* ≤ 0.01) were both significantly correlated with activity against *T. interdigitale*. For *A. australis* honey, antioxidants were significantly negatively correlated with activity against *S. aureus* (*P* ≤ 0.05) and *T. interdigitale* (*P* ≤ 0.05), suggesting that antioxidant compounds in these honeys may antagonize the effect of other active compounds. Correlations were also assessed between physical and chemical property data (Fig. 4B). For *T. hockingsi* honey, phenolic content was significantly correlated with H_2_O_2_ (*P* ≤ 0.05), color intensity (*P* ≤ 0.01), and antioxidant content (*P* ≤ 0.01). No correlations between any physical and chemical properties were seen in *T. carbonaria* or *A. australis* samples, suggesting compositional differences from *T. hockingsi* honey.

### Stingless bee honeys differ in their overall protein composition compared to honey bee honey

**Fig 5:**
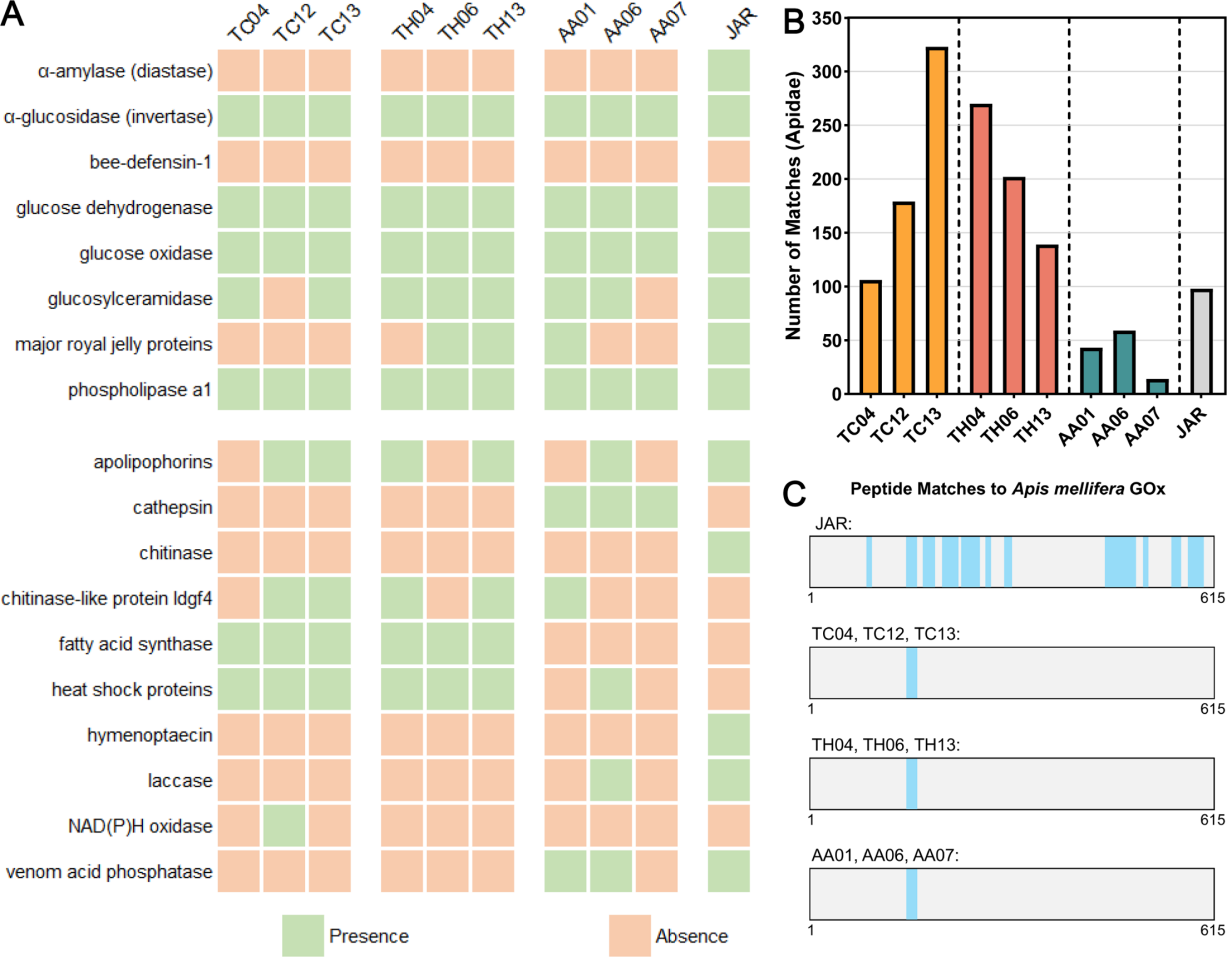
Proteins putatively identified in stingless bee honey. (**A**) The presence or absence of peptide matches to common honey proteins (top) or proteins potentially associated with antimicrobial properties (bottom) in stingless bee honey samples compared with *A. mellifera* jarrah honey (JAR). (**B**) The number of peptides matches the proteins in the Apidae database for each sample. (**C**) Peptide matches to *A. mellifera* glucose oxidase were identified in all honey samples; however, while JAR returned 19 peptide matches, the stingless bee honey samples each returned only two peptide matches.

To investigate the protein composition of stingless bee honey, three honey samples from each species (TC04, TC12, and TC13 for *T. carbonaria*; TH04, TH06, and TH13 for *T. hockingsi*; and AA01, AA06, and AA07 for *A. australis*) and *A. mellifera* jarrah honey were subjected to proteomic analysis. The presence and absence of peptide matches to common honey proteins, as well as proteins potentially associated with antimicrobial activity, are summarized in Fig. 5A, with all peptide matches to the Apidae database shown in Table S2. Common honey proteins, including α-glucosidase (invertase), glucose dehydrogenase, glucose oxidase, glucosylceramidase, and phospholipase A1, were found across honeys from all stingless bee species and in the *A. mellifera* jarrah honey. Major royal jelly proteins were detected in *T. hockingsi* (2/3 samples) honey, a single *A. australis* honey, and jarrah honey but were absent in *T. carbonaria* honey. Interestingly, α-amylase (diastase), a key enzyme typically found in honey bee honey involved in the breakdown of starches into sugars, was present only in the jarrah honey and was absent from all stingless bee honeys. Bee-defensin-1, an antimicrobial peptide, was neither detected in any of the stingless bee honey nor in the jarrah honey.

Upon manually searching for proteins potentially involved in antimicrobial activity, several key differences were observed. Apolipophorins, involved in insect immune responses, were present in the jarrah honey and in *T. carbonaria* (2/3), *T. hockingsi* (2/3), and *A. australis* (1/3) honeys. Cathepsin, a protease associated with immune function and pathogen degradation, was exclusively detected in all *A. australis* honeys (3/3). Chitinase, which breaks down chitin, a key component of fungal cell walls, was only found in the jarrah honey. Chitinase-like protein ldgf4, which may serve a similar role, was variably present in *T. carbonaria* (2/3), *T. hockingsi* (2/3), and *A. australis* (1/3) honeys.

Fatty acid synthase, an enzyme involved in lipid metabolism, was found exclusively in *T. carbonaria* (3/3) and *T. hockingsi* (3/3) honeys. Heat shock proteins, which assist in protein folding and stress responses, were consistently present in *T. carbonaria* (3/3), *T. hockingsi* (3/3) honeys, and in one *A. australis* honey but were not seen in the jarrah honey. Hymenoptaecin, an antimicrobial peptide, was detected only in the jarrah honey, while laccase, an enzyme involved in oxidative reactions and lignin degradation, was found in *A. australis* (1/3) and jarrah honeys. NAD(P)H oxidase, associated with the oxidation of phenolic compounds, was present in a single *T. carbonaria* honey, and venom acid phosphatase, typically involved in venom toxicity, was detected in *A. australis* (2/3) honey and jarrah honey.

Comparing the overall number of peptide matches to proteins in the Apidae database (Fig. 5B), *A. mellifera* jarrah honey returned 98 matches, while *T. carbonaria* and *T. hockingsi* honeys exhibited notably higher numbers of peptide matches on average (203 and 204, respectively), although there was considerable variation across the different samples. In contrast, *A. australis* honeys returned considerably fewer matches with an average of 39. This likely reflects species-specific differences in protein diversity, secretion, or preservation in honey. Peptide matches to *A. mellifera* glucose oxidase, the key enzyme responsible for H_2_O_2_ production in honey bee honey, were detected across all stingless bee and honey bee samples (Fig. 5C). However, while jarrah honey returned 19 peptide matches, stingless bee honey had only two peptide matches (GKLNGGTTLHHGMAYHR with or without oxidized methionine) across all samples, suggesting potential differences in the structure and function of this enzyme between honey bee and stingless bee honeys.

### Phenolic extracts of stingless bee honey have antibacterial and antifungal activity

**Fig 6:**
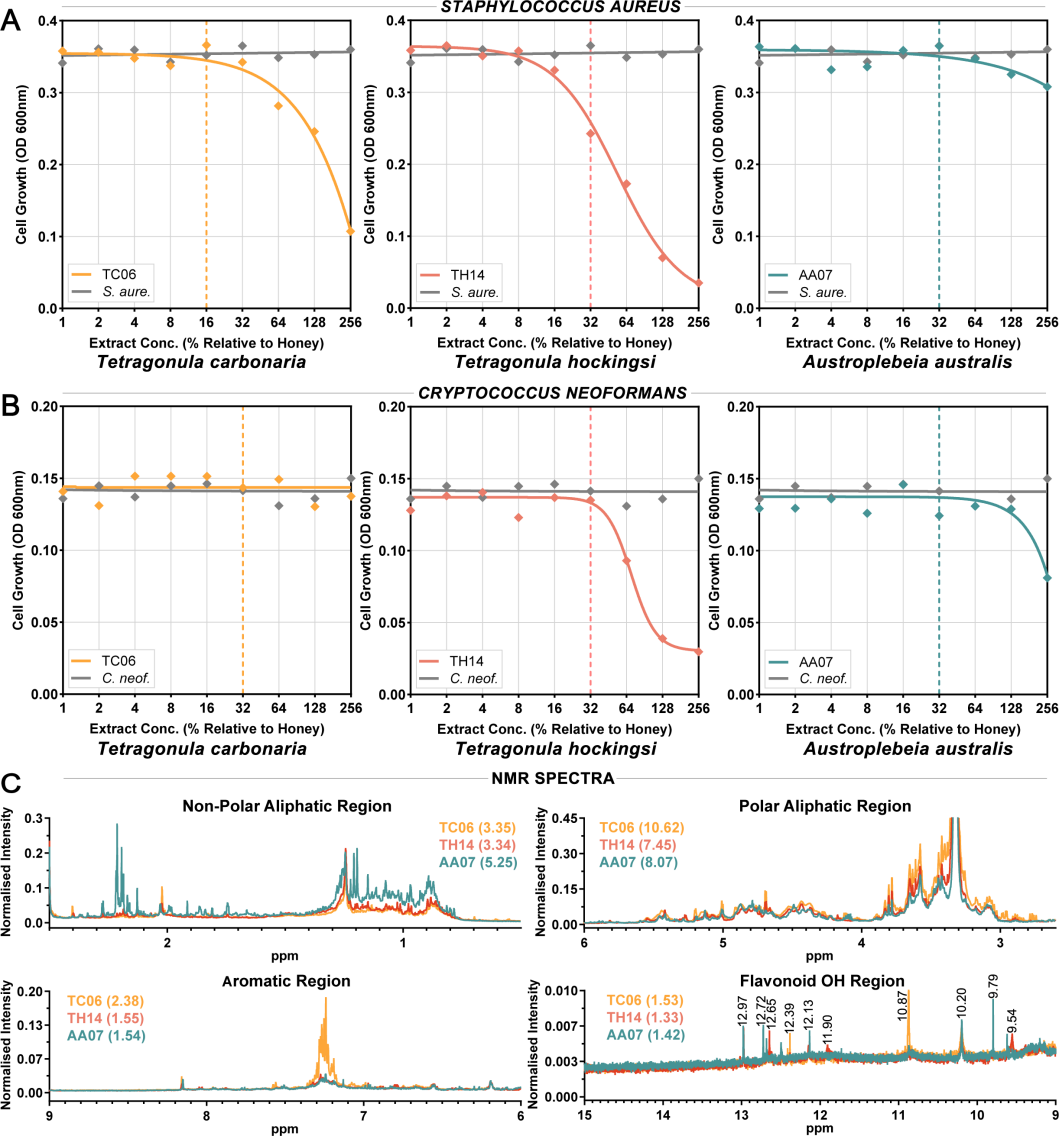
Activity of phenolic extracts of stingless bee honey against bacteria and fungi. Dose-response curves showing the inhibitory effects of phenolic honey extracts on the growth of (**A**) the bacterium *S. aureus* and (**B**) the fungus *C. neoformans*, as measured via optical density (OD) at 600 nm. Nonlinear regression lines depict the concentration-dependent responses, and diamonds show the individual values averaged across two independent replicates. The concentration of phenolic extracts is expressed as % relative to the concentration of those compounds in the original honey sample. Dashed lines indicate the non-peroxide activity MIC of the corresponding honey sample. (**C**) ^1^H NMR spectra of phenolic extracts (~5 mg/mL solutions in d^6^-DMSO, 500 MHz, and 300 K) with intensity normalized by the DMSO residue signal (2.50 ppm). Numbers in parentheses are the relative integrated intensities of the corresponding regions, where the intensity of the DMSO peak is taken as 1.0.

Based on their correlation with antimicrobial activity in *T. hockingsi* honey samples, phenolic compounds were considered a likely source of non-peroxide antimicrobial activity. Phenolic extracts were prepared for samples TC06 from *T. carbonaria*, TH14 from *T. hockingsi*, and AA07 from *A. australis* by solid-phase extraction using hydrophobic Amberlite XAD-2 resin. Approximately 18.4 g of TC06, 10.2 g of TH14, and 19.8 g of AA yielded 53, 33, and 57 mg of methanolic extract, respectively (yield 0.30 ± 0.02 mass %). Extracts were dissolved in DMSO, and their antimicrobial activity was tested using *S. aureus* and *C. neoformans*, with the concentration expressed as % relative to the concentration estimated to be present in the original honey sample (Fig. 6A and B). *T. hockingsi* sample TH14 extract was the most active, showing substantial inhibitory effects against *S. aureus* and *C. neoformans* beginning at 16% and 64%, respectively, with near-complete inhibition of both at 256%. *T. carbonaria* sample TC06 extract was active against *S. aureus*, showing increasing inhibitory effects in the range of 64%–256%, but had no activity against *C. neoformans* at the tested concentrations. *A. australis* sample AA07 extract had some limited activity against both *S. aureus* and *C. neoformans*, showing inhibitory effects against both in the range of 128%–256%. All phenolic extracts were active at concentrations that were greater than their estimated concentration in the corresponding raw honey samples at MIC.

The composition of the phenolic extracts was investigated using spectroscopic and chromatographic techniques. Figure 6C shows overlays of ^1^H NMR spectra for the three phenolic extracts, with spectra calibrated and normalized using the DMSO residual peak at 2.50 ppm (Material S3; Fig. S2). Integration of main spectral regions relative to the DMSO peak (25) showed differences in chemical composition between the samples. The *A. australis* (AA07) extract exhibited the highest abundance of non-polar aliphatic protons (0.5–2.4 ppm), indicating enrichment in fatty acids and terpenoids (26). In contrast, the *T. carbonaria* (TC06) extract exhibited the highest abundance of polar aliphatic and aromatic protons (2.6–9 ppm), indicating the presence of carboxylic acids, sugars, and aromatic compounds. In all samples, sharp singlet signals in the 9–14 ppm region, characteristic of the C_5_-OH groups of flavonoids, were detected. Some of these signals could be attributed to known flavonoids (apigenin at 12.97 ppm and pinocembrin at 12.13 ppm) (27), although most could not be definitively assigned.

Extracts were further investigated by LC-MS. The chromatograms of the samples, detected at both 260 (general aromatic compounds) and 350 nm (flavonoids) (28), showed distinct elution patterns (Material S3; Fig. S3). Notably, the *T. hockingsi* (TH14) extract exhibited the highest relative intensity at 350 nm, indicating a high flavonoid content. An intense sharp signal at 2.99 min, absent from the other samples, may contribute to the potent antimicrobial properties of the TH14 extract. Negative ion MS identified this peak as a likely flavonoid bis-glycoside, with an m/z of 593.18 agreeing with the molecular formula C_27_H_30_O_15_ (Material S3; Fig. S4). This formula corresponds to ~450 known structures of flavonoid bis-glycosides including those of apigenin, luteolin, and quercetin, according to the CAS SciFinder Chemical Substance Database.

### *T. carbonaria* honey samples remain highly active after 18 years of storage

**Fig 7:**
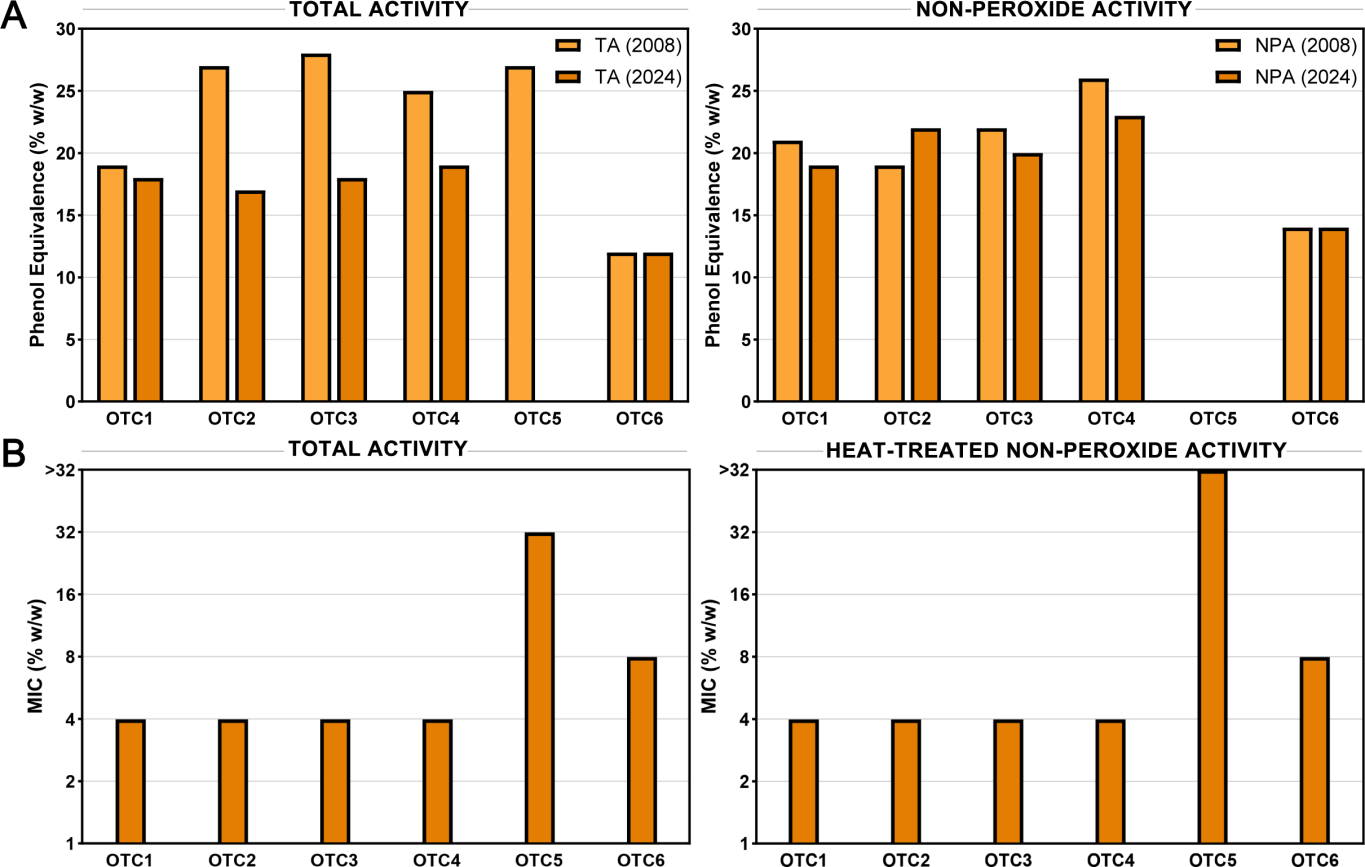
Antimicrobial activity over time in *T. carbonaria* honey samples. Antimicrobial activity against *S. aureus* with % (wt/wt) referring to the concentration of honey diluted in sterile water. (**A**) Results of the phenol equivalence assay performed in 2008 compared to 2024. For non-peroxide activity, honey was treated with a 5,600 U/mL catalase solution. (**B**) Results of the MIC assay performed in 2024. For heat-treated non-peroxide activity, honey was diluted to 64% (wt/wt), heated to 80°C for 30 min, and allowed to return to RT before testing.

The antimicrobial activity of six *T. carbonaria* honey samples previously assessed in 2008 using the phenol equivalence assay (6) and stored in the dark at 4°C was re-assessed using this same assay to determine how activity had changed over time (Fig. 7A). While total activity fell in most samples, their non-peroxide activity, measured after the honey had been treated with a catalase solution to neutralize the action of H_2_O_2_, was very stable over time. All five samples that had non-peroxide activity in 2008 showed similar levels in 2024 with no substantial differences noted. HPLC analysis confirmed that this activity was not due to MGO, with the six honeys having <12 ppm of MGO (considered negligible and below the level of detection for activity) and 0 ppm of its precursor DHA (Table S1). Honey sample OTC5, which had strong peroxide-only activity in 2008, was completely inactive in 2024. The activity of honey samples was also assessed using broth microdilution methodology to determine the MIC, with heat treatment used to deactivate glucose oxidase and destroy residual H_2_O_2_ (Fig. 7B). MIC results showed the same pattern, with no difference between total and heat-treated non-peroxide activity, except for sample OTC5 which had a low level of total activity (32% [wt/wt]) that was abolished with heat treatment.

### Microbial presence in honey samples

**Fig 8:**
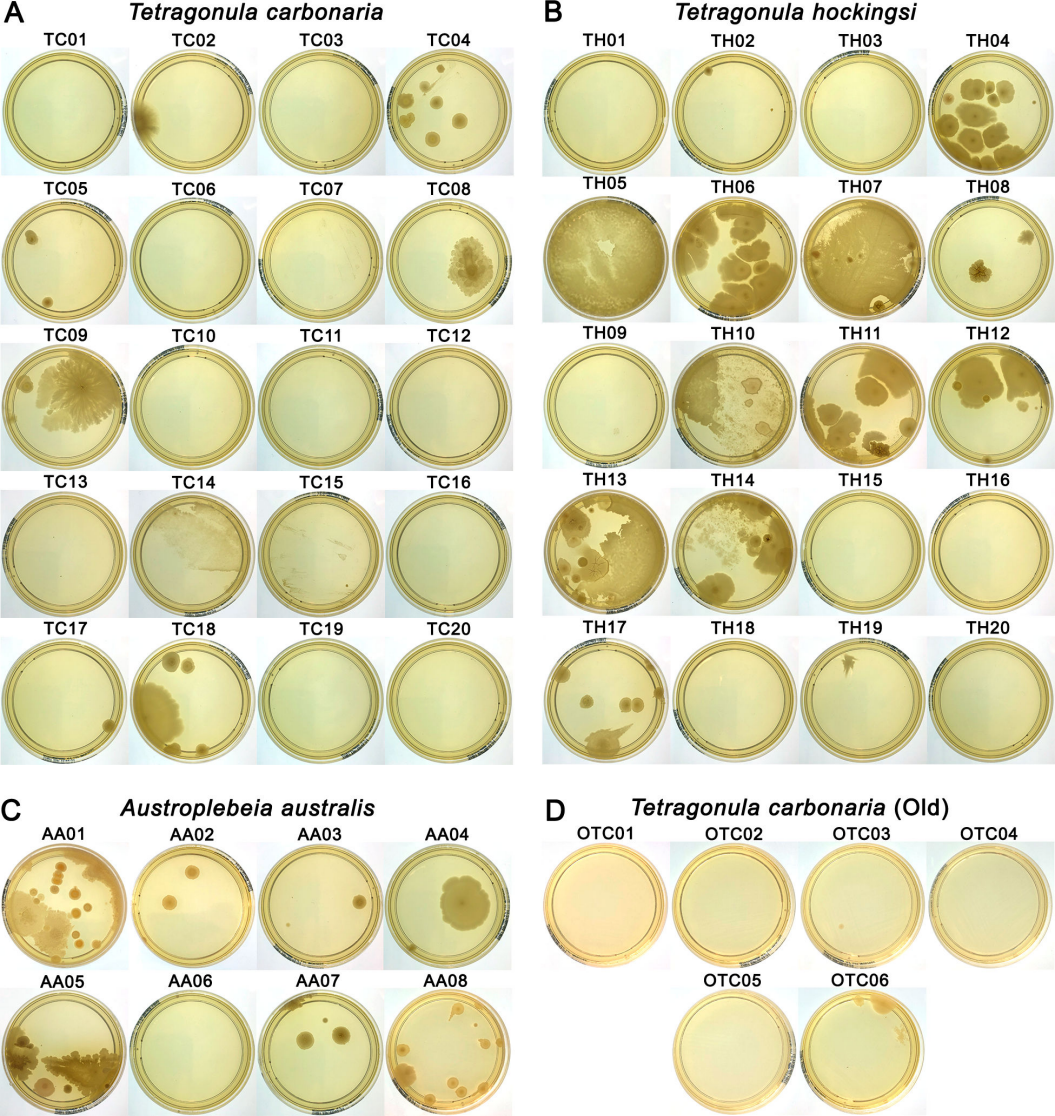
Growth of microbes from stingless bee honey samples. Spread plates of honey samples from (**A**) *T. carbonaria*, (**B**) *T. hockingsi*, and (**C**) *A. australis* after 1–2 years of storage, or (**D**) ~18 years of storage for *T. carbonaria* (old). A sterile cotton swab was dipped in undiluted honey and spread on NA plates, which were incubated at 35°C for 5 days.

The high-water content of stingless bee honey compared to *A. mellifera* honey might be assumed to support more microbial growth. All stingless bee honey samples, including those stored since 2008, were therefore spread onto agar plates to assess microbial presence. Nutrient agar plates were chosen to allow the growth of a wide range of microorganisms, and plates were imaged after incubation at 35°C for 5 days (Fig. 8). A substantial proportion of honey samples had no or minimal microbial growth. This was most evident in *T. carbonaria* honey, where 45% of honey plates had no growth, and 80% had three colonies or fewer. In *T. hockingsi,* 30% of honey plates had no growth, and 50% had three colonies or fewer. However, the remaining plates exhibited the most substantial growth across the three bee species honeys. For *A. australis* honey*,* only 13% had no growth, although 50% had three colonies or fewer. The *T. carbonaria* honeys that had been in refrigerated storage for 18 years were remarkably sterile, with 67% of honey plates having no growth and 100% having less than three colonies.

## DISCUSSION

This study provides a detailed evaluation of the antimicrobial properties and chemical profiles of honey produced by the Australian stingless bee species *T. carbonaria*, *T. hockingsi*, and *A. australis*. Our findings reveal a high antimicrobial potential of these honeys against diverse human pathogens, equivalent to or greater than that observed in most *A. mellifera* honeys. The antimicrobial properties of Australian stingless bee honey likely arise from a complex interplay of factors. These include H_2_O_2_ production, phenolic content, and other yet-to-be-identified bioactive compounds that are likely influenced by species-specific biology and environmental factors. Overall, these findings highlight the potential for stingless bee honey to be utilized in therapeutic contexts.

### Unique properties of stingless bee honey and their medical potential

A key finding of this study is the consistent presence of heat-treated non-peroxide antimicrobial activity across all stingless bee honey samples. Unlike most non-manuka *A. mellifera* honey, which primarily relys on H_2_O_2_ generated by the enzyme glucose oxidase for its antimicrobial effects (29), stingless bee honey retained significant antimicrobial properties after heat treatment (80°C for 30 min) to denature enzymes and degrade H_2_O_2_, with some *T. carbonaria* and *T. hockingsi* honey MICs remaining as low as 8% (wt/wt) against *S. aureus* and *E. coli* (Table 1; Fig. 1). This feature is particularly relevant for medical applications, ensuring sustained antimicrobial efficacy even when H_2_O_2_ degrades over time or due to environmental factors such as heat or light. The persistence of activity after heat treatment further suggests the potential for heat-based sterilization in medical applications. Notably, *T. carbonaria* honeys tested after 18 years of storage retained their non-peroxide activity with no significant decrease, highlighting the long-term stability of this activity (Fig. 7).

Another notable finding in stingless bee honey is the exceptionally high level of H_2_O_2_ production, particularly in *T. carbonaria* honey, which reached over 100 ppm and continued for at least 6 days in some samples (Fig. 3). Proteomic analysis revealed only two peptide matches to honey bee glucose oxidase in the stingless bee honey (Fig. 5) compared to 19 matches for the *A. mellifera* jarrah honey. This suggests potential structural or functional differences in glucose oxidase between these bee species and may explain the much stronger and prolonged production of H_2_O_2_ observed in stingless bee honey.

This prolonged production could offer significant advantages for wound dressings and other medical applications that require sustained antimicrobial action (30). A gradual release of H_2_O_2_ over time would minimize the need for frequent reapplications, a particularly advantageous feature in high microbial load environments. Additionally, stingless bee honeys were found to be high in antioxidants (Table 1; Fig. 2), with *T. carbonaria*, *T. hockingsi*, and *A. australis* honeys having an average antioxidant content of 8,467, 8,632, and 4,848 µmol Fe^2+^/kg, respectively, compared to a notably lower average of 2,329 µmol Fe^2+^/kg that has been observed for diverse Australian *A. mellifera* honeys (19). This could further enhance their wound-healing potential by mitigating oxidative stress while maintaining antimicrobial efficacy (31). With their broad-spectrum activity against bacterial and fungal pathogens, the dual action of high H_2_O_2_ production and heat-treated non-peroxide activity in *T. carbonaria* and *T. hockingsi* honeys makes them promising candidates for clinical use.

### Sources of antimicrobial activity in stingless bee honeys

HPLC analysis confirmed the absence of significant MGO in stingless bee honey (Table S1). Unlike manuka honey, where non-peroxide activity is derived from the properties of the nectar of *Leptospermum* plants (32), the consistent presence of heat-treated non-peroxide activity in stingless bee honey across diverse locations suggests that bee-related factors play a critical role. This hypothesis is further supported by the occurrence of non-peroxide activity in honey from various stingless bee species worldwide, including *Trigona* species in Malaysia (11), *Scaptotrigona* species in Brazil (12), and *Melipona* and *Hypotrigona* species in Nigeria (33). This implies that bee-derived proteins, peptides, or metabolites are responsible for this activity, and/or these bees selectively forage on plants rich in specific bioactive compounds. Notably, sharp singlet signals of flavonoids in the 9–14 ppm range of ^1^H NMR spectra have shown a strong correlation with the anti-microbial and anti-inflammatory properties of propolis (26, 34). This supports the theory of selective foraging as well as the possibility that bioactive compounds may transfer from propolis storage pots into the honey, as was previously suggested for *T. carbonaria* (9). Together, these findings suggest that the antimicrobial properties of stingless bee honey are shaped by an interplay of bee-specific biology, foraging behaviors, and nest-associated ecological factors, similar to what is observed in *A. mellifera* (35).

While all stingless bee honeys displayed strong antimicrobial activity, investigating their physical and chemical properties revealed notable species-specific differences (Fig. 2). Compared to our previous study using a collection of 30 Australian *A. mellifera* honeys from diverse flora (19), stingless bee honeys across all species had higher average H_2_O_2_ production at 1–2 h (22 vs 48 ppm, respectively) and greater antioxidant content (2,329 vs 7932 µmol Fe^2+^/kg, respectively). *A. australis* honey had a similar average phenolic content (154 mg GAE/kg) to *A. mellifera* honey (146 mg GAE/kg), while *T. carbonaria* and *T. hockingsi* honeys showed much higher levels (257 and 366 mg GAE/kg, respectively). Conversely, the average pH of *T. carbonaria* (3.64) and *T. hockingsi* (3.81) honeys was were close to those of *A. mellifera* honey (3.72), whereas *A. australis* (4.13) honey was notably higher. The observed values for phenolic content, antioxidant activity, and pH align with ranges found in previous studies on *T. carbonaria* and *T. hockingsi* honey (36).

Correlations between antimicrobial activity and physical and chemical properties were also different across species (Fig. 4). The antimicrobial activity of *T. carbonaria* honey was strongly correlated with H_2_O_2_ production, while in *T. hockingsi* honey, this was only seen in the inhibition of the fungal dermatophyte *T. interdigitale*, which is known to be highly susceptible to peroxide stress (37). In *T. hockingsi* honey, color intensity was correlated with antibacterial activity and phenolic content with activity against *T. interdigitale*, with the active TH14 phenolic extract exhibiting high flavonoid glycoside content (Fig. 6). *T. carbonaria* honey, in addition to H_2_O_2_ activity, had strong heat-treated non-peroxide activity, and sample TC06 had an antibacterial phenolic extract, despite its relatively low phenolic content. Notably, NMR data indicated that TC06 had a higher overall aromatic content than TH14, consistent with the aromatic compounds in TC06 being highly polymerized and therefore inactive (38). *A. australis* honey was different again, showing high total activity but low heat-treated non-peroxide activity, with antioxidant activity negatively correlated with activity against *S. aureus* and *T. interdigitale* and a minimally active phenolic extract produced from sample AA07. These findings suggest that honey from each stingless bee species exhibits distinctive mechanisms of antimicrobial action that could potentially be exploited against specific pathogens or for specific purposes. For example, the antioxidant activity present in the *T. carbonaria* and *T. hockingsii* honeys could be beneficial for treating inflamed tissues or in wound healing (39). Further exploration with a broader panel of microbes would provide deeper insights into these species-specific honey properties.

Our proteomic analysis of the different honey samples revealed highly diverse matches with proteins in the Apidae database and variable presence of proteins with potential antimicrobial functions. For example, in *A. australis* honey, proteins such as cathepsin (involved in protein degradation), laccase (associated with oxidation reactions and lignin degradation), and venom acid phosphatase (involved in venom processing) were detected. If these play a role in antimicrobial activity, this could account for the substantial reduction in activity observed in heat-treated *A. australis* honey. A major limitation of these results is the lack of proteomic databases for stingless bee species, meaning that most peptide matches were made to *Apis* and *Bombus* species, and antimicrobial peptides and proteins unique to Meliponini may have been missed. As proteomic databases become more complete for stingless bees, revisiting this data set could provide deeper insights into bioactive compounds present in stingless bee honey.

### Scalability of stingless bee honey production for medicinal use

Scaling up the production of stingless bee honey for medicinal use presents a challenge, primarily due to the limited honey yields per colony. Stingless bee colonies produce honey in much smaller quantities than *A. mellifera*, with individual hives yielding around 0.5 L or less per year, depending on the species (3). This low yield is partly due to the smaller size of Australian stingless bees, which limits their per-colony honey production compared to larger stingless bee species found overseas. Unlike *A. mellifera*, Australian stingless bee species are native and not cultivated outside the country. According to Halcroft in 2013, annual sugar bag honey production in Australia is estimated at just a few hundred kilograms, far lower than that of overseas industries in countries such as Mexico where larger stingless bee species allow small cooperatives to produce 500–1,500 kg each annually (4). While stingless beekeeping in Australia remains largely a hobby, interest has grown significantly over the past two decades, with hive numbers steadily rising (40). In particular, more beekeepers are keeping large numbers of stingless bee hives for commercial use as crop pollinators (41). Given that factors like floral source diversity (15) and hive health (42) can affect the chemical composition of honey, it will be essential to focus on improving hive management practices in addition to scaling up hive numbers to transform stingless bee honey into a viable commercial product. Recent legislative changes also support this potential, with native stingless bee honey now approved by Food Standards Australia New Zealand, opening the door for national and international commercialization (43).

Storage is another challenge for the scalability of stingless bee honey. In our recent study, *A. mellifera* honeys stored in the dark at 4°C for over 15 years showed an average 64% decrease in peroxide-based activity, yet still retained significant peroxide-based antimicrobial potential (19). In contrast, in the current study, *T. carbonaria* honeys stored in the dark at 4°C for 18 years completely lost any peroxide-based activity, although their non-peroxide activity remained intact. These long-term storage experiments highlight key differences in stability, likely tied to the higher moisture content of stingless bee honey. We also show that while some stingless bee honeys were remarkably sterile, others, particularly *T. hockingsi* honey, have the potential to support significant microbial growth (Fig. 8), which may accelerate the degradation of antimicrobial compounds. Gamma irradiation, which is widely used on medicinal *A. mellifera* honey without significant degradation of activity (44, 45), could be used to sterilize medicinal stingless bee honey, and its effect on the activity should be explored. Freezing is an alternate preservation method that has been previously shown to prevent degradation of glucose oxidase in royal jelly over 1 year (46) and could help maintain safety and bioactivity over time, particularly if the honey is used as a functional food and cannot be gamma irradiated.

### Conclusion

This study demonstrates the strong and consistent antimicrobial potential of Australian stingless bee honeys, particularly their heat-stable non-peroxide activity, positioning them as promising candidates for medical use. Further research identifying specific bioactive compounds, including phenolic and antioxidant compounds, proteins, and peptides unique to stingless bee species, will be key to unlocking their full therapeutic potential.

## SUPPLEMENTAL MATERIAL

Table S1 aem.02523-24-s0001.xlsx All activity, physical, and chemical property data.

Table S2 aem.02523-24-s0002.xlsx All proteomic data.

Material S3 aem.02523-24-s0003.docx Supplemental methods and Fig. S1 to S4.

ASM does not own the copyrights to Supplemental Material that may be linked to, or accessed through, an article. The authors have granted ASM a non-exclusive, world-wide license to publish the Supplemental Material files. Please contact the corresponding author directly for reuse.
