# Supplementary material for: Strong antimicrobial activity and unique physicochemical characteristics in honey from Australian stingless bees Tetragonula carbonaria, Tetragonula hockingsi, and Austroplebeia australis
Source: Appl Environ Microbiol. 2025 May 21;91(6):e02523-24. doi: 10.1128/aem.02523-24 (PMC12175512; doi:10.1128/aem.02523-24)
Supplement: Material S3 — Supplemental methods and Fig. S1 to S4. [file aem.02523-24-s0003.docx]

**Supplementary Material S3**

**Isolation and Characterization of Stingless Bee Honey Phenolic Extracts**

Phenolic fractions of honeys from *Tetragonula carbonaria* (sample TC06), *Tetragonula hockingsii* (sample TH14) and *Austroplebeia australis* (sample AA07) were isolated by solid phase extraction with hydrophobic Amberlite XAD-2 resin (Merck Cat. No. 10357), using a modified literature procedure [S1]; the experimental workflow is shown in Figure S1.

Figure S2 shows overlays of ^1^H NMR spectra of phenolic extracts (~5 mg mL^−1^ in d^6^-DMSO). The spectra were collected on a Bruker Avance 500 MHz spectrometer at 300 K, using a standard ^1^H NMR setup (a sum of 64 scans), and processed by MestReNova Lite-14.0.0-23239 (Mestrelab Research, Santiago de Compostela, Spain) and OriginPro 2022 (OriginLab, Northampton, MA, USA) software. The spectra were calibrated and normalized using the DMSO residual peak at 2.50 ppm, as shown in Figure S2. Integration of main spectral regions [S2] relative to the DMSO peak has shown that the sample AA07 had the highest abundance of non-polar aliphatic protons (0.5-2.4 ppm, Figure 6C in the main text), while the sample TC06 had the highest abundance of polar aliphatic and aromatic protons (2.6-6 ppm and 6-9 ppm, respectively). These data suggest that the TC06 extract is enriched in carboxylic acids, sugars and aromatic compounds, while the AA07 extract is enriched in fatty acids and terpenoids [S3].

Appearance of sharp singlet signals of C_5_-OH groups of flavonoids in the 9-14 ppm region of ^1^H NMR spectra has been described for propolis extracts dissolved in d^6^-DMSO [S3, S4]. The presence of these signals showed a strong correlation with the anti-microbial and anti-inflammatory properties of propolis [S3, S4]. Such signals were also observed for the phenolic extracts of stingless bee honeys (Figure 6C in the main text), although their intensity was low compared to typical propolis extracts [S3, S4]. Some of the signals could be attributed to specific flavonoids (apigenin at 12.97 ppm and pinocembrin at 12.13 ppm) [S5], although most of the signals could not be identified.

The LC-MS analysis of phenolic extracts was performed using a Waters Acquity system. Solid phenolic fractions were dissolved at ~2 mg mL^−1^ in H_2_O-MeCN mixture (1 : 1 vol/vol) that contained ~0.5 M NH_3_, then filtered through a 0.22 μm membrane filter. The use of NH_3_ assisted the deprotonation of carboxylic acid and phenol groups within the sample and increased the intensity of negative ion MS signals, since most of the components of phenolic fractions were previously identified in the negative ion mode [S1, S6]. Aliquots of the filtrates (5.0 μL) were injected into a Sunrise UPLC C_18_ column and eluted for 10 min using 100% H_2_O to 100% MeCN gradient with 0.20 mL min^−1^ flow rate. UV-vis spectra of the eluants were collected at the 220-500 nm range with a PDA detector, and negative ion MS were collected at the 150-800 m/z range (capillary voltage, 0.8 kV).

A comparison of elution profiles for the samples TC06, TH14 and AA07 at 260 nm (general aromatic groups) and 350 nm (flavonoids) [S7] is shown in Figure S3. The profiles show the presence of multiple sharp peaks at 2-4 min on the top of a broad unresolved signal, which is likely due to the presence of polymerization products [S8]. Overall relative intensity of the 350 nm over the 260 nm signal increased in the order AA07 < TC06 < TH14 (Figure S3), which was consistent with the changes in total phenolics content for the corresponding honeys (Figure 2 in the main text). In particular, the TH14 sample showed an intense sharp signal in the 350 nm profile at 2.99 min, which was absent for the other samples (Figure S3). The UV-vis spectrum corresponding to this signal (Figure S4A-B) showed a maximum at 265 nm and a shoulder at ~350 nm, which are typical for flavonoids and their derivatives [S7]. The corresponding negative ion MS signal was observed at m/z = 593.18 (Figure S4C), which agrees with the molecular formula C_27_H_30_O_15_ (MW = 594.15845) for the neutral species [S9]. This formula corresponds to ~450 known structures of bis-glycosides of flavonoids (apigenin, kaempherol, luteolin, quercetin etc.) [S10], one of which is illustrated in Figure S4D. The presence of flavonoid glycosides in phenolic extracts of stingless bee honey is consistent with literature data [S6]. High degree of flavonoid glycosylation can also explain the low intensity of signals due to C_5_-OH protons in the ^1^H NMR spectra (Figure 6C in the main text).

In summary, the high anti-bacterial and anti-fungal activity of the TH14 extract (Figure 6A-B in the main text) can be attributed to the high content of flavonoid glycosides, while the low activity of the AA07 extract can be due to the prevalence of fatty acids and terpenoids. The TC06 extract was relatively poor in phenolics (Table 1 in the main text) and showed relatively low antimicrobial activity (Figure 6A-B) despite the high total content of aromatic compounds determined by ^1^H NMR spectroscopy (Figure 6C). This can be explained by high degree of polymerization of phenolic compounds [S8] that decreased the content of active phenolic groups.


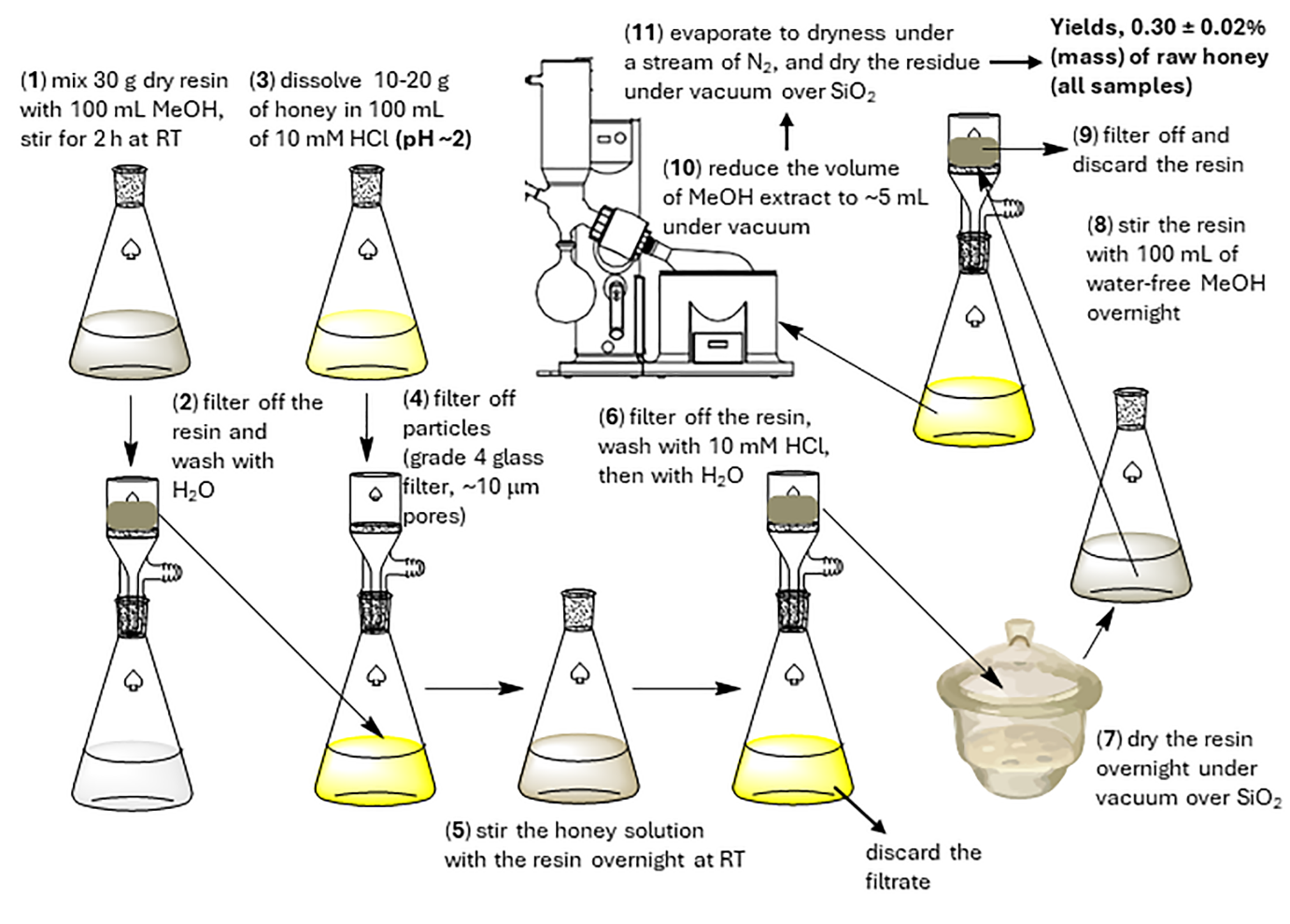


**Figure S1.** Workflow for solid phase extraction of phenolics from stingless bee honey, modified from a literature method [S1]. The resin is Amberlite XAD-2 (Merck Cat. No. 10357).


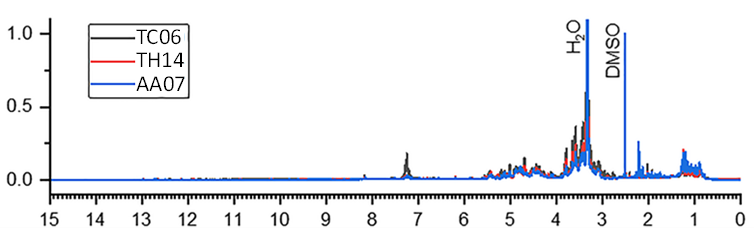


**Figure S2.** ^1^H NMR spectra of phenolic extracts (~5 mg mL^−1^ solutions in d^6^-DMSO, 500 MHz, 300 K); intensity normalized by the DMSO residue signal (2.50 ppm).


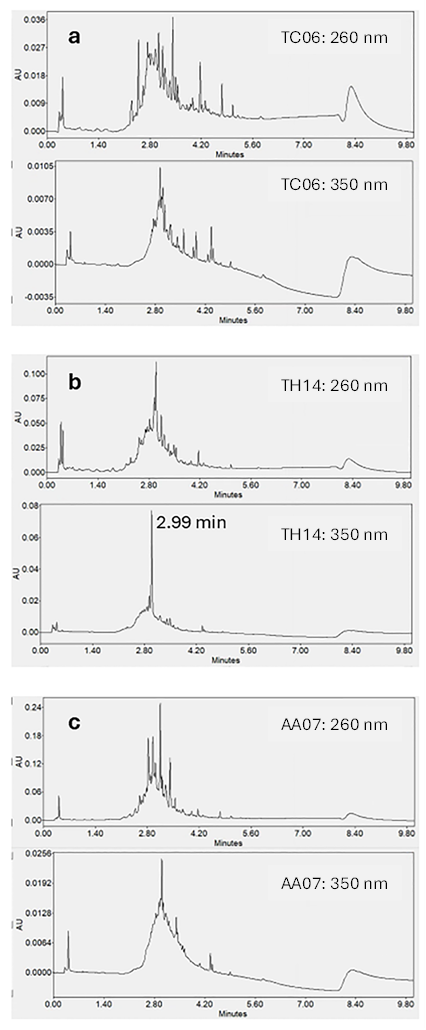


**Figure S3**. UV-vis elution profiles at 260 nm and 350 nm for LC-MS analysis of phenolic extracts (~2 mg mL^−1^ in 1:1 H_2_O-MeCN, containing ~0.5 M NH_3_).


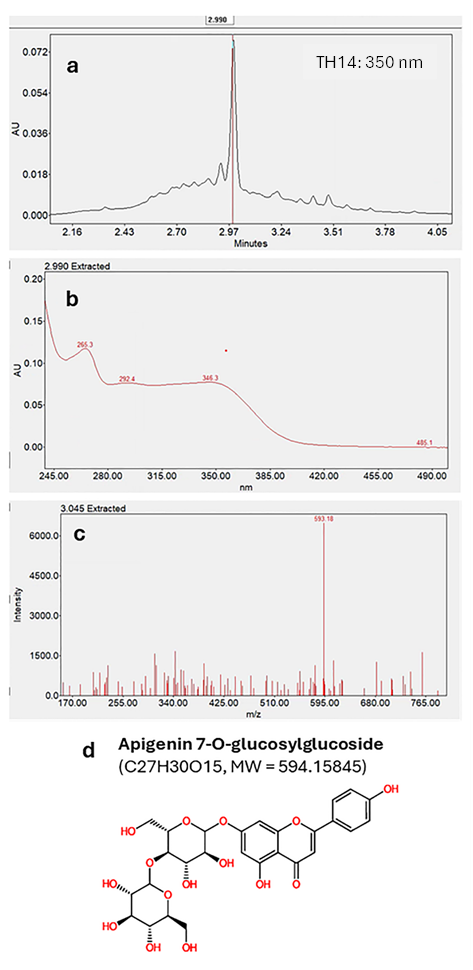


**Figure S4.** Details of LC-MS analysis for the TH14 extract: (**a**) close view of the 2.99 min peak in the elution profile at 350 nm; (**b**) full UV-vis spectrum for the 2.99 min peak; (**c**) negative ion mass spectrum corresponding to the 2.99 min peak (the time offset between the MS and UV-Vis data is 0.05 min); and (**d**) one of the possible structures of flavonoid bis-glycosides, corresponding to the m/z = 593.18 signal [S2, S3].

**Supplementary References**

[S1] Kassim, M.; Achoui, M.; Mustafa, M. R.; Mohd, M. A.; Yusoff, K. M., Ellagic acid, phenolic acids, and flavonoids in Malaysian honey extracts demonstrate in vitro anti-inflammatory activity. *Nutrition Research* **2010,** *30* (9), 650-659. Doi 10.1016.j.nutres.2010.08.008.

[S2] Li, Y.; Harir, M.; Lucio, M.; Kanawati, B.; Smirnov, K.; Flerus, R.; Koch, B. P.; Schmitt-Kopplin, P.; Hertkorn, N., Proposed guidelines for solid phase extraction of Suwannee river dissolved organic matter. *Analytical Chemistry* **2016,** *88* (13), 6680-6688. Doi 10.1021/acs.analchem.5b04501.

[S3] Tran, C. T. N.; Brooks, P. R.; Bryen, T. J.; Williams, S.; Berry, J.; Tavian, F.; McKee, B.; Tran, T. D., Quality assessment and chemical diversity of Australian propolis from *Apis mellifera* bees. *Scientific Reports* **2022,** *12* (1), 13574. Doi 10.1038/s41598-022-17955-w.

[S4] Rivero-Cruz, J. F.; Rodríguez de San Miguel, E.; Robles-Obregón, S.; Hernández-Espino, C. C.; Rivero-Cruz, B. E.; Pedraza-Chaverri, J.; Esturau-Escofet, N. Prediction of antimicrobial and antioxidant activities of Mexican propolis by ^1^H-NMR spectroscopy and chemometrics data analysis. *Molecules*, **2017**, *22*, 1184. Doi 10.3390/molecules22071184.

[S5] Papotti, G.; Bertelli, D.; Plessi, M.; Rossi, M. C., Use of HR-NMR to classify propolis obtained using different harvesting methods. *International Journal of Food Science & Technology* **2010,** *45* (8), 1610-1618. Doi 10.1111/j.1365-2621.2010.02310.x.

[S6] Massaro, C. F.; Shelley, D.; Heard, T. A.; Brooks, P., In vitro antibacterial phenolic extracts from “sugarbag” pot-honeys of Australian stingless bees (*Tetragonula carbonaria*). *Journal of Agricultural and Food Chemistry* **2014,** *62* (50), 12209-12217. Doi 10.1021/jf5051848.

[S7] Taniguchi, M.; LaRocca, C. A.; Bernat, J. D.; Lindsey, J. S., Digital Database of Absorption Spectra of Diverse Flavonoids Enables Structural Comparisons and Quantitative Evaluations. *Journal of Natural Products* **2023,** *86* (4), 1087-1119. Doi 10.1021/acs.jnatprod.2c00720.

[S8] Latos-Brozio, M.; Masek, A.; Piotrowska, M., Polymeric forms of plant flavonoids obtained by enzymatic reactions. *Molecules* **2022,** *27* (12), 3702. Doi 10.3390/molecules27123702.

[S9] MassBank Europe (https://massbank.eu/), accessed July 2024.

[S10] CAS SciFinder Chemical Substance Database (<https://www.cas.org/solutions/cas-scifinder-discovery-platform/cas-scifinder>), accessed July 2024.
